# The application of a single session of capacitive resistive electric transfer 24 h before exercise modifies the accelerometric pattern in standardbred racing trotters

**DOI:** 10.1186/s12917-024-04039-2

**Published:** 2024-05-22

**Authors:** David Argüelles, Aritz Saitua, Raquel Miraz, Natalie Calle-González, Francisco Requena, Irene Nocera, Valentina Vitale, Micaela Sgorbini, Ana Muñoz

**Affiliations:** 1 Department of Animal Medicine and Surgery, Faculty of Veterinary Medicine, University of Córdoba, Córdoba, Spain; 2 Equine Sport Medicine Center CEMEDE, School of Veterinary Medicine, University of Córdoba, Córdoba, Spain; 3 Department of Cellular Biology, Physiology and Immunology, School of Veterinary Medicine, University of Córdoba, Córdoba, Spain; 4 Department of Veterinary Sciences, University of Pisa, Pisa, Italy; 5 Department of Animal Medicine and Surgery, School of Veterinary Medicine, Cardenal Herrera-CEU University, Valencia, Spain

**Keywords:** Accelerometry, Capacitive resistive electric transfer, Exercise, Horse, Locomotion, Training

## Abstract

**Background:**

It has been reported that capacitive resistive electric transfer (CRET) increases blood circulation, hemoglobin oxygenation and temperature in muscles. The attributed benefits of these changes have been linked to improved athletic performance, enhanced muscle flexibility and fastening recovery from exercise-induced fatigue. For all of this, the present research aims to investigate whether the application of CRET 24 h before exercise affects the accelerometric pattern in horses during exercise. Six sound Standardbred trotters were subjected to a CRET session of 40 min of duration, applied on both sides of the neck, back and croup, 24 h before a training session. Training sessions consisted of a warming-up (WU) for 6400 m and a training bout (TB) at their maximal training speed for 1600 m. The same protocol was followed for the device off (sham protocol), also applied 24 h before the training session. CRET and sham experiments were separated by one week, the order of application of both was randomly defined for each individual and drivers were blinded for the duration of the experiment. During the training sessions, horses wore an accelerometer fixed at the sternal level. Speed, stride frequency (SF), length (SL), regularity and symmetry and accelerometric activities were measured during WU and TB.

**Results:**

CRET increased speed, mediolateral and total accelerometric activities during WU and speed, SL, dorsoventral, longitudinal and total accelerometric activities during TB, but stride regularity and symmetry decreased.

**Conclusion:**

The application of CRET 24 h before exercise increased speed and accelerometric activities, results that highlight the need to evaluate the interaction between CRET and training in order to develop new methods to limit fatigue. However, the decrease in stride regularity and symmetry after CRET application could be negative effects, which could be attributed to the increased speed.

## Background

Radiofrequency is within the electromagnetic spectrum, in the range from 3 kHz to 300 GHz [1, 2]. In recent years, several companies have developed systems with frequencies between 0.3 MHz (300 kHz) and 1 MHz. While these systems operate in the radiofrequency range, the application of energy is done by direct contact with the patient’s skin. The most common range of electromagnetic currents is between 400 and 450 kHz (448 kHz). Because this technique uses capacitive and resistive electrodes, it is called capacitive resistive electric transfer, or CRET and it is considered a non-invasive electrothermal therapy.

The physiological effects have been associated with two large groups of actions: electrical and thermal. It has been demonstrated that intermittent exposure to a 448 kHz electric stimulus, as used in the electrothermal CRET therapies, up-regulates cellular signaling pathways, promoting proliferation in mesenchymal stem cells [3–5]. Furthermore, these in vitro research have suggested that CRET application might promote tissue regeneration by activating the proliferation of the stem cells present in damaged areas [3–5]. These effects have been considered direct consequences of the application of the electrical currents, occurring even at low intensity, with minimal heat accumulation. Moreover, CRET currents could increase the temperature in target organs due to the electrical resistivity of the tissues, by transferring energy without introducing radiant energy from the exterior [6, 7]. Consequently, CRET enables an endothermic effect when the applied intensity is moderate to high and, depending on the impedance offered by the tissues upon passage of the currents. Despite this thermal effect, tissue hyperthermia can be avoided by dissipating heat by the circulating blood towards the adjacent areas [8, 9].

The endothermic effect results in increased deep and superficial blood circulation, and oxyhemoglobin levels and improvement in joint range of motion, flexibility, and functional movements [8]. This effect as a performance-enhanced tool during exercise and training has been suggested by Duñabeitia et al. [10], who described that a CRET session applied after intense exercise resulted in less evident biomechanical changes attributable to muscle fatigue and consequently, they hypothesized that this therapy might accelerate recovery after intense exercise in human recreational runners. More recently, Wachi et al. [11] asserted that 4 min of CRET therapy applied as a passive warm-up therapy in calf muscles of healthy men increased muscle temperature in a shorter time compared to traditional active warm-up techniques (stretching and jogging for 4 min) and resulted in increased jump performance. The mean rise of temperature from basal values, measured at a depth of 10 mm, was of 2.0 and 1.4ºC for CRET and active warm-up respectively [11].

Locomotor pattern is closely related to performance in the equine athlete, and it has been widely studied by accelerometry in horses competing in different disciplines [12, 13]. Accelerometry is a kinetic method that measures instantaneous changes in velocity derived from applying a force to an object or body [14]. Small sensors measure acceleration of the surface to which they are attached, and the acceleration vector obtained is proportional to the force applied to the body in which it has been fixed, providing an objective, practical, reliable, and low-cost technique to evaluate locomotion in horses [14].

We have already studied how CRET application affects the accelerometric pattern of horses exercised at fixed walking and trotting velocities on a treadmill [2]. Our results showed that the application of CRET resulted in increased total accelerometric activity (TAA), which triggered a longer stride length (SL). Because the horses’ speed was kept unchanged on the treadmill, the horses showed a reduction in stride frequency (SF) after the CRET application, and this effect persisted for 24 h after CRET application. The physiological basis of these changes is not completely elucidated at this moment and requires deeper studies. However, to the author’s best opinion, these changes in the gait pattern could be due to the thermal effects of CRET. Additionally, increased longitudinal or propulsive accelerometric activity (LAA) and stride regularity (REG) were also found. Because LAA represents the amount of acceleration in the craniocaudal axis, a relationship with speed has been described [12, 15]. On the contrary, lower LAA has been associated with a decrease in speed when various types of sedatives are administered [16]. The positive relationship between LAA and speed was justified because longitudinal acceleration is associated with the propulsive work of a moving horse.

As the accelerometric pattern of the horse depends on the type of exercise performed, the present investigation analyzes the accelerometric pattern in Standardbred trotters during a regular training session, on two different occasions: 24 h after the application of a CRET session, and 24 h after the application of the same session but with the machine off (sham procedure). Our investigation aimed to elucidate whether and how the accelerometric pattern is modified with the application of a CRET session in trotters. We hypothesized that: firstly, the application of CRET would result in increased TAA, derived mainly from an increase in LAA, causing an increase in SL; and secondly, the REG and symmetry (SYM) of the stride would also be improved.

## Results

During the warming-up (WU) after the CRET application, horses had significantly greater speed (*p* = 0.021), mediolateral accelerometric activity (MLAA) (*p* = 0.010) and TAA (*p* = 0.014) (Table 1). Cliff’s δ revealed a small size effect on REG (decrease), SYM (decrease), DVAA (increase), LAA (decrease), MLAA (increase) and TAA (increase) during the WU.

**Table 1:** Median and 25–75% quartiles of the locomotor parameters measured by accelerometry in Standardbred trotters in two experiments: sham (24 h after application of a capacitive resistive electrical transfer session with the machine off) and CRET (24 h after application of a capacitive resistive electrical transfer session), during the warming-up of a routine training session (* significant differences at *p* < 0.05)

	Sham	CRET	*P*	Cliff’s δ
Speed (m/s)	6.40 (5.00-7.50)	6.90 (7.28–7.28)	0.021 *	-0.062
SF (strides/s)	1.66 (1.61–1.76)	1.71 (1.61–1.81)	0.550	-0.050
SL (m/stride)	3.86 (3.01–4.34)	4.03 (2.92–4.04)	0.374	-0.015
REG (dimensionless)	329.0 (314.0-348.0)	326.0 (292.0-345.0)	0.390	0.172 #
SYM (dimensionless)	200.0 (179.0-227.0)	184.0 (171.0-184.0)	0.073	0.198 #
DVD (cm)	6.0 (6.0–7.0)	7.0 (5.0–8.0)	0.178	-0.091
DVAA (W/kg)	20.2 (16.1–23.7)	20.7 (18.4–20.7)	0.126	-0.172 #
LAA (W/kg)	9.9 (8.7–11.9)	9.8 (8.8–13.4)	0.268	0.125 #
MLAA (W/kg)	9.0 (7.0-12.7)	12.8 (7.5–15.2)	0.010 *	-0.215 #
TAA (W/kg)	37.8 (32.2–48.9)	45.0 (34.0-52.5)	0.014 *	-0.204 #

(SF: stride frequency; SL: stride length; REG: regularity; SYM: symmetry; DVD: dorsoventral displacement; DVAA: dorsoventral accelerometric activity; LAA: longitudinal accelerometric activity; MLAA: mediolateral accelerometric activity; TAA: total accelerometric activity)

Small (#) effect size changes of CRET compared to sham procedure according to Cliff’s δ effect size thresholds

During the training bout (TB), also after CRET application, significantly greater values for speed (*p* = 0.000), SL (*p* = 0.000), dorsoventral accelerometric activity (DVAA (*p* = 0.000), LAA (*p* = 0.020) and TAA (*p* = 0.049) were found. The maximal individual speed increased from 9.50 to 10.65 m/s. On the contrary, lower values for REG (*p* = 0.004) and SYM (*p* = 0.000) were found after CRET application during the TB (Table 2). Small size effects for REG (decrease) and DVD (increase); moderate size effects for SF (decrease), SYM (decrease), DVAA (increase) and MLAA (increase); and large size effects for velocity (increase), SL (increase), LAA (increase) and TAA (increase) were found according to Cliffs’ δ (Table 2),

**Table 2:** Median and 25–75% quartiles of the locomotor parameters measured by accelerometry in Standardbred trotters in two experiments: sham (24 h after application of a capacitive resistive electrical transfer session with the machine off) and CRET (24 h after application of a capacitive resistive electrical transfer session), during the training bout of a routine training session (* significant differences at *p* < 0.05)

	Sham	CRET	*p*	Cliff’s δ
Speed (m/s)	9.50 (9.03–10.15)	10.65 (10.0-11.65)	0.000 *	-0.611 †
SF (strides/s)	2.00 (1.90–2.10)	1.95 (1.90–2.05)	0.302	0.417 §
SL (m/stride)	4.59 (4.50–5.26)	5.68 (4.74–5.80)	0.000 *	-0.570 †
REG (dimensionless)	278.5 (245.3–306.0)	242.5 (213.5-272.3)	0.004 *	0.285 #
SYM (dimensionless)	209.0 (176.5-233.3)	165.0 (145.0-182.3)	0.000 *	0.379 §
DVD (cm)	5.0 (4.0–6.0)	5.0 (4.3-6.0)	0.230	-0.193 #
DVAA (W/kg)	34.4 (30.9–38.9)	35.8 (30.2–44.6)	0.000 *	-0.369 §
LAA (W/kg)	31.9 (25.1–48.9)	41.7 (32.9–56.3)	0.020 *	-0.512 †
MLAA (W/kg)	30.9 (24.8–38.4)	34.2 (24.0-41.3)	0.661	-0.431 §
TAA (W/kg)	100.9 (85.8-116.7)	111.7 (93.8-132.7)	0.049 *	-0.680 †

(SF: stride frequency; SL: stride length; REG: regularity; SYM: symmetry; DVD: dorsoventral displacement; DVAA: dorsoventral accelerometric activity; LAA: longitudinal accelerometric activity; MLAA: mediolateral accelerometric activity; TAA: total accelerometric activity)

Small (#), moderate (§) and large (†) effect size changes of CRET compared to sham procedure according to Cliff’s δ effect size thresholds

When the accelerometric activities were expressed as a percent of the TAA, an increase in MLAA% was found during WU (*p* = 0.045), but a reduction during TB in the CRET experiment (*p* = 0.000). During the TB, a greater LAA% was found in the CRET experiment (*p* = 0.000) (Table 3).

**Table 3:** Median and 25–75% quartiles of the accelerometric activities in Standardbred trotters in two experiments: sham (24 h after application of a capacitive resistive electrical transfer session with the machine off) and CRET (24 h after application of a capacitive resistive electrical transfer session), expressed as percentages of the total accelerometric activity TAA, during the warming-up and during the training bout of a routine training session (* significant differences at *p* < 0.05)

	Sham	CRET	*p*
Warming-up			
DVAA (%TAA)	49.6 (47.2–52.2)	48.9 (43.4–53.4)	0.635
LAA (%TAA)	25.2 (22.6–28.4)	26.1 (20.4–29.9)	0.120
MLAA (%TAA)	23.4 (21.5–27.1)	27.7 (21.7–29.8)	0.070 *
Training bout			
DVAA (%TAA)	33.1 (30.6–35.5)	32.9 (26.6–37.4)	0.780
LAA (%TAA)	34.6 (29.7–39.8)	37.9 (34.4–40.6)	0.020 *
MLAA (%TAA)	32.9 (26.1–38.6)	28.5 (25.5–31.6)	0.000 *

(DVAA: dorsoventral accelerometric activity; LAA: longitudinal accelerometric activity; MLAA: mediolateral accelerometric activity; TAA: total accelerometric activity)

Correlations between the different locomotor parameters measured in the two experiments, sham and CRET, are presented in Tables 4 and 5. Positive correlations were found for speed and accelerometric activities in both experiments. Neither experiment correlated speed with DVD. Significant negative correlations of speed with stride REG and SYM were also observed.

**Table 4:** Spearman correlation (*p* values within brackets) of the locomotor parameters measured by accelerometry in Standardbred trotters during a routine training session (including warming-up and training bout), performed after 24 h of a capacitive resistive electrical transfer session with the device off (sham experiment)

	SF	SL	REG	SYM	DVD	DVAA	LAA	MLAA	TAA
S	0.726 (0.000)	0.928 (0.000)	-0.583 (0.000)	-0.012 (0.877)	-0.396 (0.000)	0.912 (0.000)	0.849 (0.000)	0.876 (0.000)	0.932 (0.000)
SF		0.429 (0.002)	-0.383 (0.000)	0.049 (0.524)	-0.721 (0.000)	0.614 (0.000)	0.657 (0.000)	0.706 (0.000)	0.703 (0.000)
SL			-0.584 (0.000)	-0.009 (0.903)	-0.206 (0.008)	0.883 (0.000)	0.794 (0.000)	0.810 (0.000)	0.880 (0.000)
REG				0.023 (0.766)	0.081 (0.296)	-0.652 (0.000)	-0.678 (0.000)	-0.617 (0.000)	-0.656 (0.000)
SYM					-0.156 (0.043)	-0.085 (0.272)	-0.024 (0.755)	-0.027 (0.731)	-0.055 (0.482)
DVD						-0.232 (0.002)	-0.371 (0.000)	-0.380 (0.000)	-0.353 (0.000)
DVAA							0.882 (0.000)	0.880 (0.000)	0.964 (0.000)
LAA								0.867 (0.000)	0.936 (0.000)
MLAA									0.939 (0.000)

(S: speed; SF: stride frequency; SL: stride length; REG: regularity; SYM: symmetry; DVD: dorsoventral displacement; DVAA: dorsoventral accelerometric activity; LAA: longitudinal accelerometric activity; MLAA: mediolateral accelerometric activity; TAA: total accelerometric activity)

**Table 5:** Spearman correlation (*p* values within brackets) of the locomotor parameters measured by accelerometry in Standardbred during a routine training session (including warming-up and training bout), performed after 24 h of a capacitive resistive electrical transfer session (CRET experiment)

	SF	SL	REG	SYM	DVD	DVAA	LAA	MLAA	TAA
S	0.71 (0.000)	0.881 (0.000)	-0.820 (0.000)	-0.550 (0.000)	-0.316 (0.000)	0.803 (0.000)	0.811 (0.000)	0.836 (0.000)	0.867 (0.000)
SF		0.494 (0.000)	-0.759 (0.000)	-0.506 (0.000)	-0.686 (0.000)	0.598 (0.000)	0.912 (0.000)	0.835 (0.000)	0.849 (0.000)
SL			-0.657 (0.000)	-0.444 (0.000)	0.001 (0.993)	0.834 (0.000)	0.590 (0.000)	0.710 (0.000)	0.746 (0.000)
REG				0.505 (0.000)	0.302 (0.001)	-0.667 (0.000)	-0.799 (0.000)	-0.722 (0.000)	-0.763 (0.000)
SYM					0.380 (0.000)	-0.293 (0.001)	-0.472 (0.000)	-0.371 (0.000)	-0.402 (0.000)
DVD						-0.026 (0.774)	-0.516 (0.000)	-0.304 (0.001)	-0.321 (0.000)
DVAA							0.670 (0.000)	0.812 (0.000)	0.846 (0.000)
LAA								0.855 (0.000)	0.898 (0.000)
MLAA									0.978 (0.000)

(S: speed; SF: stride frequency; SL: stride length; REG: regularity; SYM: symmetry; DVD: dorsoventral displacement; DVAA: dorsoventral accelerometric activity; LAA: longitudinal accelerometric activity; MLAA: mediolateral accelerometric activity; TAA: total accelerometric activity)

## Discussion

Capacitive resistive electric transfer, a radiofrequency technique at 448 kHz that promotes the physiological processes of tissue metabolism by transferring energy, has been increasingly used in human exercise physiology and sports medicine during the last 20 years. Although at the beginning, this electrotherapy technique was predominantly used for the treatment of musculoskeletal disorders, in the last years, however, CRET has been applied in an attempt to improve sports performance, delay or limit fatigue associated with training and increase the elasticity/flexibility of various musculoskeletal tissues including tendons, ligaments or muscles.

Our previous research demonstrated that accelerometric activities increased in horses exercised on a treadmill at defined walk and trot velocities [2]. Total accelerometric activity reached the highest values between 2 and 26 h after the application of CRET. Although the increase in TAA led to significant changes in the amount of acceleration distributed towards the three body axes, when DVAA, LAA, and MLAA were expressed as a percent of TAA, DVAA decreased, but LAA increased. Based on these previous results, in the present study, we firstly hypothesized that the application of CRET would increase TAA and particularly LAA. In main lines, our hypothesis was confirmed, even though a significant increase in LAA was not found during WU after CRET application. Large size effects were detected in LAA during TB but small size effects during WU. We cannot scientifically and reliably demonstrate the reason for these increases in accelerometric activity, but we could propose that there might be a direct consequence of muscle hyperthermia, since CRET is classified as a deep diathermy technique. A mild increase in muscle temperature, as occurs during active or passive warm-up before an exercise, causes a series of positive physiological consequences: decreased muscle stiffness of muscle fibers during contraction; increased oxygen delivery to muscles via a right-ward shift in the oxyhemoglobin dissociation curve and vasodilation of muscle blood vessels, increased in oxygen uptake and use in the muscles and enhanced aerobic energy transduction by accelerating the rate-limiting reactions associated with oxidative phosphorylation [17, 18]. A strong association between power output and muscle temperature, either passively or actively increased, has been established in humans, with a 1ºC increase in muscle temperature being shown to enhance subsequent exercise performance by 2–5%, depending on the type and speed of the muscle contractions. The proposed mechanisms appear to increase ATP turnover and cross-bridge rate improvements in muscle fiber functionality and muscle fiber conduction velocity [19, 20]. Additionally, the magnitude of the muscle temperature response has been positively associated with the speed of the movement [19].

At this moment, the amount of increased temperature in deep muscles or other tissues after different protocols of CRET application in horses is unknown. We have conducted a pilot study to measure this increase in temperature, but only non-invasive procedures have been used, measuring the skin temperature in the thoracolumbar region of the horse [21]. Mean increases of 7ºC and 12ºC in skin temperature were found after the application of 5 min of capacitive at 40% and 10 min of resistive therapy at 40%, reaching maximal temperatures of 33.0 ºC (32.7–36.1 ºC- lower and upper quartiles). However, as these changes have been measured in the skin, not in the deep musculature, further investigations regarding the effects of the deep musculature are needed to confirm these hypotheses. Erasala et al. [22] demonstrated that an increased tissue temperature of 38ºC, 40ºC and 42ºC (measured in the trapezius muscle of healthy humans) corresponded to increased local blood flow of respectively 27, 77 and 144%. In horses, Haussler et al. [23] assumed that temperatures applied to the skin should be 38–43ºC for heating therapeutic modalities. Even if these high temperatures were not reached during the CRET application, as indicated before, a rise in 1ºC determines an increase of 10–15% in local tissue metabolism, enhancing oxygenation and removing toxic products [24] and this effect may underlie the locomotor changes.

We applied CRET technique 24 h before a training session. To the authors’ knowledge, the duration of the hyperthermia on the muscles after application has not been determined and the proper time to reach the best effect is unknown at this moment. Kumaran and Watson [25] measured the skin thermal response after CRET administration in the lower thigh region of adult humans and found that the higher temperature was sustained for at least the first 45 min of follow-up. Temperatures after this time were not measured. In the same way, Tashiro et al. [8] measured the temperature at the skin surface and at depths of 10 and 20 mm in 13 healthy males after CRET application in the lower paraspinal muscle and the temperature in the three sites persisted elevated until the end of the experiment, at 30 min. Our research group found that at 30 min after CRET application in the thoracolumbar region of healthy horses, temperature persisted elevated over the baseline values [21]. At this time, we do not know if muscle temperature remains elevated beyond the first 45 min after application or if, alternatively, its effects persist even if the increase in temperature dissipates.

If we combine the results obtained in human runners [10] and horses [2, 26], the locomotory modifications persist during exercise when the technique is administered 24 h before. As discussed previously, we do not know if these results might be attributable to increased muscle temperature and/or to the secondary effects of this hyperthermia (i.e. increased blood flow, increased collagen extensibility, increased oxyhemoglobin…), or if they are a secondary effect of the electrical currents, with a consequent recovery of the action potentials of the muscle fibers. Consequently, we could hypothesize that the CRET application in our study could have enhanced oxygenation and hastened recuperation of the muscle from previous training sessions, resulting in an improvement of the accelerometric pattern.

Regardless of the mechanism of action, the present study revealed an increase in TAA during a training session conducted 24 h after CRET application compared to the sham experiment, as well as positive correlations between TAA and LAA and between LAA and speed. Size effect evaluated with Clifff’s δ showed a negligible effect of CRET application in LAA and TAA during the WU, but this size effect was large during the TB. A possible explanation for these results could be that the riders controlled the speed more during the WU than during the TB, where the horses were able to develop a greater speed. Barrey et al. [12] stated that the longitudinal acceleration was linked to the propelling work of running, because the longitudinal (or cranio-caudal) acceleration resulted from the restraint and propulsive forces of the hooves on the ground. However, when comparing Standardbred trotters with different performances, a correlation between the LAA and the performance index was not found, even though significantly higher values for LAA were found in the intermediate and high-performance groups compared to the low performance group [15]. It is plausible to think that a greater amount of acceleration in the longitudinal axis would increase SL, this being another hypothesis of our research. Another interesting result was the increased percentage of the TAA directed towards the MLAA during exercise after CRET application, which was observed both during WU and TB, suggesting greater flexibility [2].

As opposed to what we expected, REG and SYM did not increase after CRET application and consequently, our second hypothesis was rejected. In fact, a significant reduction was found during TB and size effects were found to be small to moderate for both parameters. A weak negative, but significant correlation was described between REG and SYM and speed in trotters by Barrey et al. [27]. These findings were attributed to orthopedic disorders or laterality in the trot. In our case, the clinical examination did not reveal any locomotor disorder or evident alteration of movement that could justify the reduction of REG and SYM. The effect of laterality of movement cannot be ruled out, neither the effect of the age, since we have studied young animals. Perhaps the increase in speed could lead to a less regular and less symmetrical gait. At this moment, we do not know the consequences of these modifications and if they will change with training and age. However, in a previous study conducted on sound adult horses, we found that REG increased after CRET at walk, but not at trot, and SYM experienced a reduction at trot, without significant changes at walk [2]. In this research, the speed was kept constant, because it was performed on a treadmill. Consequently, there must be other factors in addition to speed involved in the reduction of REG and SYM after CRET application, which need to be further evaluated.

We would like to point out that this is a preliminary study, in which a small number of animals have been included, since we tried to select a group of horses as homogeneous as possible in terms of training levels, training techniques and management procedures. Even so, the results have been consistent in magnitude and direction for all recruited horses. It would be advisable to conduct the study with a larger number of animals, of different ages and levels of training, to verify the influence of CRET on sports performance and the interactions with training adaptations. There are many unknown facts about this technique in the field of sport performance that must be unraveled before a more extended application.

Although there is previous data, the number of investigations regarding the relationship between accelerometric variables, measured both during a locomotory test and during racing, with performance is scarce. In our research, we have applied CRET 24 h before exercise because in previous research we found that this was the time when accelerometric changes were most evident [2]. The timing of the application of CRET before exercise might be critical and the effect of different periods/ protocols of applications have not been evaluated yet. A recent article has evaluated the effects of CRET therapy as a warm-up strategy before exercise [28]. Although the authors documented its usefulness as a procedure to reduce muscle stiffness, they did not evaluate its effect on performance and recommended an active warm-up afterward. The application of CRET as a passive warm-up strategy, followed by an active warm-up period should be evaluated in horses from various types of sporting disciplines.

Although the temperature increase caused by this technique can be beneficial before exercise, a great increase in muscle temperature can also be detrimental. Wallace et al. [29] pointed out that when there is a great increase in core temperature, fatigue increases and the athlete’s performance decreases. This study also highlighted how the psychological perception of heat can negatively affect sports performance [29]. Furthermore, we have used the same application protocol as Becero et al. [2], but other protocols should be evaluated on horses performing different types of exercises and in interaction with a training program. We have not found any negative side effects associated with the CRET therapy, although in all cases, it was applied by an experienced equine clinician. Special caution must be taken into consideration if the therapy is applied by inexperienced professionals with the technique, in order to avoid the negative effects of hyperthermia.

## Conclusions

The application of a CRET session 24 h before exercise, compared with the same technique applied with the device off, significantly modifies the accelerometric pattern, inducing significant increases in accelerometric activities during exercise. However, the regularity and symmetry of the stride decreased. The interactions of the application of this radiofrequency technique with training must be studied. Likewise, the use of this technique as a passive warm-up strategy before exercise or to promote a better and faster recovery after exercise should also be investigated in depth in the near future.

## Methods

### Materials

This research has been approved by the Ethical Committee for Animal Welfare of the Veterinary Clinical Hospital of the University of Córdoba (Ref: 28/2018; date of approval: June 21st, 2018; Title: The effect of capacitive resistive electrical transfer on sport horses’). The owner or person in charge of the horses was informed about the procedure, objectives, possible benefits and risks of the study and they signed the agreement accepting the inclusion of their horses in this research.

Six sound Standardbred trotting horses (2 geldings and 4 mares), with a mean age of 3.17 years (range 3–4 years) from the same training stable were recruited. All of them were in active training for at least one year before the study. Horses were competing at the same level in races of one mile (1600 m). The training protocol was the same for all the horses because they had the same trainer.

The following criteria were used to select the horses: (1) young Standardbred horses aged between 3 and 4 years, in active training during the last year before enrolling in the study; (2) to have the same competitive level and training (selected by the trainer; no objective data to prove training level); (3) to be in active competition; (4) to hold the study protocol, with the corresponding interventions and evaluations and not be subjected to any other treatment or intervention; (5) to be sound at walk and at trot, over different surfaces and conditions, i.e. not to have clinically evident lameness according to the grading scale of the American Association of Equine Practitioners (AAEP); (6) to have no pathological findings on cardiac and respiratory auscultation and no complaints of performance loss.

### Capacitive resistive electrical transfer device and application

Capacitive resistive electrical transfer (CRET) was applied with the device Indiba® Animal Health Vet905 (Indiba® S.A., Barcelona, Spain). It operates at a frequency of 448 kHz. A rigid circular metal electrode was used as the active electrode (diameter: 35 and 65 mm, according to the area), and a large flexible rectangular metallic plate (size: 200 × 260 mm) was used as inactive electrode. The device releases radiofrequency energy in two modes at the active electrode: capacitive (CAP) and resistive (RES). The CAP electrode has a polyamide coating that acts as a dielectric medium, insulating its metallic body from the skin surface and generating superficial heat near the skin. The RES electrode is uncoated, and therefore, radiofrequency energy travels directly through the body into the inactive electrode, generating heat in the deeper parts of the body. The inactive electrode was placed on the sternum during CRET applications. More information concerning this device has been previously published [1, 2].

**Fig. 1:**
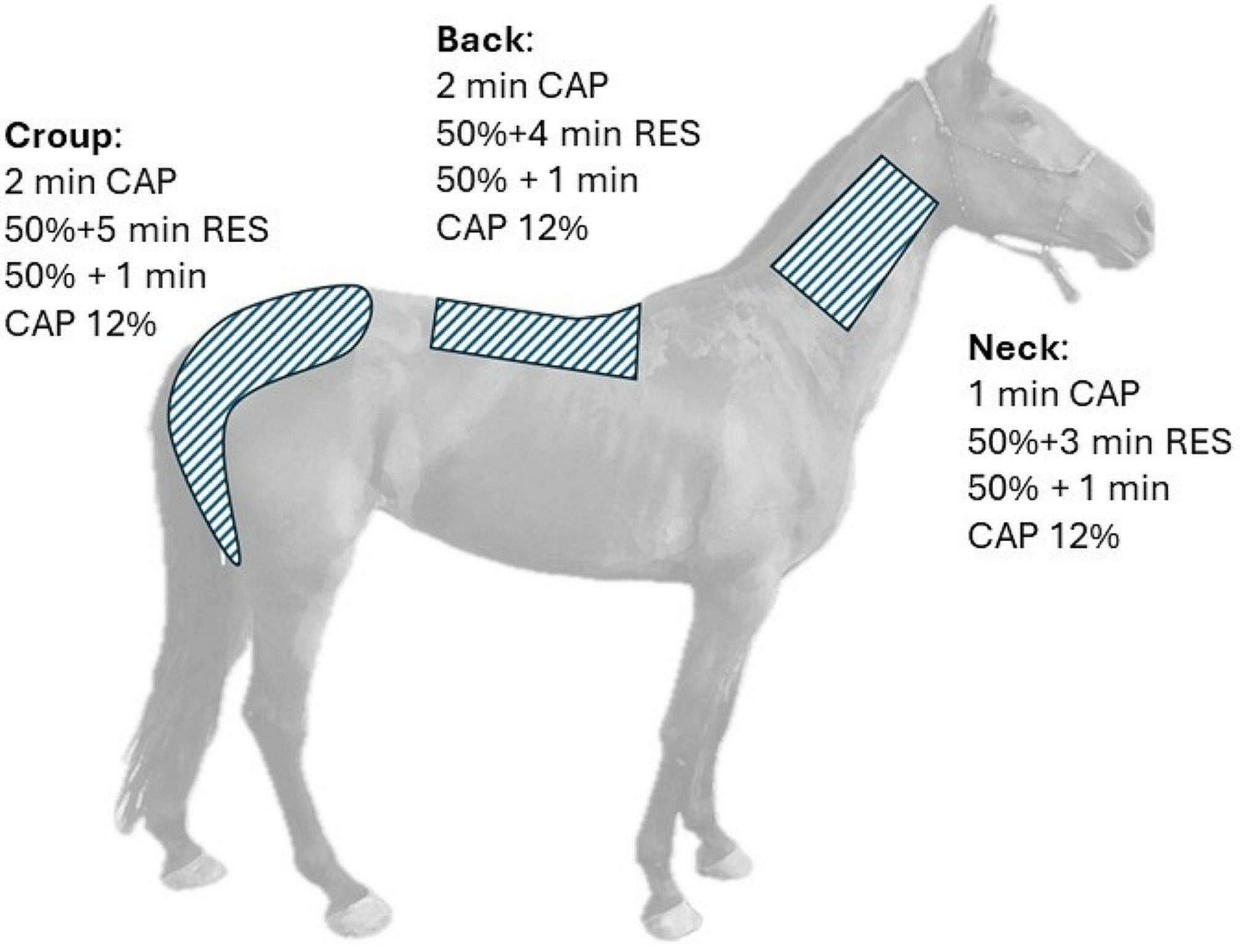
Representation of the anatomical areas and protocols used during the sham and CRET experiments

Sessions had a total duration of 40 min, 20 min per side of the horse. The therapy was applied on the epaxial musculature of the neck, back and croup always in a craniocaudal direction, as indicated in Fig. 1. Therapy was applied to the right side first in 3 horses and to the left side first in the remaining 3, alternating between the two protocols, sham and CRET. Firstly, CAP at 50% intensity was applied (1 min neck, 2 min back and 2 min croup), followed by RES at 50% intensity (3 min neck, 4 min back and 5 min croup) and at the end, CAP at 12% intensity (1 min neck, 1 min back and 1 min croup). This protocol was similar to those applied in our previous studies, where we found that CRET application triggers significant changes on locomotion in horses evaluated on a treadmill at fixed walking and trotting speeds [2] and during a dressage test [26], although it was shortened due to logistical considerations.

### Experimental design

Two experiments were performed: a control exercise session (performed 24 h after the CRET session with the device off, named sham) and the same exercise 24 h after CRET at the intensities previously described (named CRET experiment). Both experiments were separated by one week, and the order of both was randomly established for each individual horse. Two different drivers participated in the study, even though the same driver drove the same horse in both experiments. Drivers and trainers were blinded by each of the experiments (i.e., sham vs. CRET).

Horses were exercised at their usual training track, with a length of 800 m, completing a total distance of 8000 m. The training session started with a warm-up period (WU), in which they trotted at a medium speed (between 6 and 7 m/s), selected by their driver, for 6400 m. After, horses had a recovery period by walking for 10 min before starting the training bout (TB), which consisted of covering 1600 m at their maximal training speed.

### Accelerometer device and data acquisition

Accelerometric data was obtained with a portable gait analyzer (Equimetrix, Centaure-Metrix®, France), consisting of three orthogonal accelerometers that measure acceleration along the three body axes, a data logger, and a scientific software program (Equimetrix-Centaure 3D®) for processing acceleration signals. The recorder collected data continuously at a sampling rate of 100 Hz.

The accelerometer was placed in the caudal part of the sternum, between the right and left *pectoralis ascendens* muscles, at the level of the girth, as previously recommended [12–14]. In this location, the device is near the body’s center of gravity, has good stability against the body of the horse, and provides more information about the acceleration parameters in the forelimb. The same researcher (A.S.) positioned the transducer on all occasions. More information about this device has been previously reported [1, 2, 12–16].

The accelerometric evaluations were performed during WU and TB, at half the exercise time in both experiments, in a straight line of 500 m.

### Locomotor parameters

Three measurements were obtained for the locomotor parameters for WU and TB, for each individual horse and in each of the experiments (sham vs. CRET) and the means were calculated, so we had a value for each horse in each experiment and each exercise bout (WU and TB). These mean values were used for statistical analysis.

The parameters measured with the accelerometer were divided into three groups: accelerometric activities and parameters, stride coordination parameters and stride spatiotemporal parameters. Accelerometric activities and parameters included dorsoventral displacement (DVD, cm), dorsoventral accelerometric activity (DVAA, W/kg), longitudinal accelerometric activity (LAA, W/kg), mediolateral accelerometric activity (MLAA, W/kg) and the sum of these last accelerometric activities, i.e., total accelerometric activity (TAA, W/kg). Besides, DVAA, LAA and MLAA were expressed as a percentage of the TAA. Stride coordination parameters included regularity, REG (dimensionless), and symmetry (SYM, (dimensionless). Stride spatiotemporal parameters included speed (S, m/s), measured with a GPS system, SF (cycles/s or Hz), and SL (m).

The definition of each of these parameters and the methods of calculation by the accelerometer are summarized in Table 6.

**Table 6:** Accelerometric parameters measured in the present study, units, definition, and calculation

Variable and units	Definition	Calculation
Accelerometric variables		
Dorsoventral displacement, DVD, cm	Displacement of the sternum (near to the center of gravity, where the accelerometer was fixed) in a dorsoventral direction	Calculated as an estimation of the double integration of the dorsoventral acceleration signal
Dorsoventral accelerometric activity DVAA, W/kg	Acceleration in the dorsoventral axis. It represents the dorsoventral activity of suspension and loading of the limbs.	Calculated as the integral of the power spectrum obtained by Fast Fourier Transformation from the dorsoventral acceleration signal
Longitudinal accelerometric activity LAA, W/kg	Acceleration in the craniocaudal or longitudinal axis. It measures the amount of acceleration and deceleration along the longitudinal axis. It is related to the braking and propulsive power	Calculated as the integral of the power spectrum obtained by Fast Fourier Transformation from the longitudinal acceleration signal
Mediolateral accelerometric activity MLAA, W/kg	Acceleration in the lateral axis. It measures the amount of acceleration and deceleration along the lateral axis	Calculated as the integral of the power spectrum obtained by Fast Fourier Transformation from the lateral acceleration signal
Total accelerometric activity TAA, W/kg	Total accelerometric activity	Calculated as the sum of the dorsoventral, longitudinal and mediolateral accelerometric activities
Coordination variables		
Stride regularity REG (dimensionless)	Similarity of successive strides over the course of time in the accelerometric pattern	Calculated as a correlation coefficient corresponding to a peak in the autocorrelation function of the dorsoventral acceleration, measured at a time equal to stride duration
Stride symmetry SYM (dimensionless)	Similarity between the left and the right acceleration patterns	Calculated as the correlation coefficient corresponding to the peak of the autocorrelation function of the dorsoventral acceleration measured at a time equal to half stride duration
Spatiotemporal variables		
Speed, m/s		Measured with a GPS
Stride frequency SF, strides/s	Number of strides per unit of time and inverse of stride duration	Calculated by detecting the frequency of the major peak of the power spectrum calculated by the Fast Fourier Transformation of the dorsoventral acceleration signal
Stride length SL, m/stride	Distance covered in a stride	Calculated by dividing the speed by the SF

### Statistical analysis

The data are presented as median and 25–75% quartiles. Kolmogorov-Smirnov and Levene’s tests were used to assess the normal distribution of the data and the homogeneity of the variance, respectively. A non-parametric Wilcoxon test was performed to compare sham vs. CRET experiments, both during the WU and TB. Cliff’s δ was calculated providing an effect size to identify thresholds for small (0.15–0.33), moderate (0.33–0.47) and large (0.47) practical data. This calculates the proportion of overlap between data of both situations (sham vs. CRET), in which a Cliff’s δ of 1 or -1 indicates no overlap and 0 represents a complete overlap between the two procedures The following thresholds have been applied: δ < 0.147, negligible effect; 0.147 < δ < 0.330, small effect; 0.331 < δ < 0.474, medium effect; δ > 0.474, large effect [30].

The relationships between the different accelerometric parameters for both the WU and TB experiments were investigated with a Spearman test correlation.

The statistical analysis was performed with the IBM SPSS Statistics 21. The level of significance was set at *p* < 0.05. Cliff’s δ values for size effects were calculated with the following calculator: https://cliffdelta.shinyapps.io/calculator [30].

## Data Availability

The authors confirm that the main supporting data of this research are available within the article. The complete raw data are available from the main author (David Argüelles) and from the corresponding author (Ana Muñoz) under reasonable request.

## Abbreviations

CAP: Capacitive
CRET: Capacitive resistive electric transfer
DVD: Dorsoventral displacement
LAA: Longitudinal accelerometric activity
MLAA: Mediolateral accelerometric activity
REG: Stride regularity
RES: Resistive
SF: Stride frequency
SL: Stride length
SYM: Stride symmetry
TB: Training bout
TTA: Total accelerometric activity
WU: Warming-up
